# Development of Artificial Cell Models Using Microfluidic Technology and Synthetic Biology

**DOI:** 10.3390/mi11060559

**Published:** 2020-05-30

**Authors:** Koki Kamiya

**Affiliations:** Division of Molecular Science, Graduate School of Science and Technology, Gunma University, 1-5-1 Tenjin-cho, Kiryu City, Gunma 376-8515, Japan; kamiya@gunma-u.ac.jp

**Keywords:** microfluidics, synthetic biology, artificial cell model, minimal cell, giant lipid vesicles, liposomes, membrane proteins, molecular robots

## Abstract

Giant lipid vesicles or liposomes are primarily composed of phospholipids and form a lipid bilayer structurally similar to that of the cell membrane. These vesicles, like living cells, are 5–100 μm in diameter and can be easily observed using an optical microscope. As their biophysical and biochemical properties are similar to those of the cell membrane, they serve as model cell membranes for the investigation of the biophysical or biochemical properties of the lipid bilayer, as well as its dynamics and structure. Investigation of membrane protein functions and enzyme reactions has revealed the presence of soluble or membrane proteins integrated in the giant lipid vesicles. Recent developments in microfluidic technologies and synthetic biology have enabled the development of well-defined artificial cell models with complex reactions based on the giant lipid vesicles. In this review, using microfluidics, the formations of giant lipid vesicles with asymmetric lipid membranes or complex structures have been described. Subsequently, the roles of these biomaterials in the creation of artificial cell models including nanopores, ion channels, and other membrane and soluble proteins have been discussed. Finally, the complex biological functions of giant lipid vesicles reconstituted with various types of biomolecules has been communicated. These complex artificial cell models contribute to the production of minimal cells or protocells for generating valuable or rare biomolecules and communicating between living cells and artificial cell models.

## 1. Introduction

Lipid vesicles or liposomes possess a phospholipid bilayer that has the same composition as that of living cells [1,2]. Although the thickness of the phospholipid bilayer in both living cells and giant vesicles is only 5 nm approximately, it separates the intracellular and extracellular environments. The phospholipid bilayer of living cells, the cell membrane, has various types of membrane proteins like ion channels [3,4], transporters [5,6], enzymes [7,8], and receptors [9,10]. These membrane proteins on the cell membrane facilitate the transport of molecules between the intracellular and extracellular environments, energy exchange [11,12], and signal transduction [13,14]. Therefore, the surface of the cell membrane plays an important role in cellular functions including the development of critical diseases, metabolism, and homeostasis.

Lipid vesicles have been used in drug delivery systems and artificial cell models. Nano-sized lipid vesicles (50–200 nm in diameter) carrying drugs like anticancer drugs or small interfering (si)RNA have been exploited as drug delivery systems [15,16,17]. Cell-sized lipid vesicles (>5 μm in diameter) are easily formed by the hydration of lipid films. They are easily observed using an optical microscope and possess the same biophysical properties as those of living cell membranes. Therefore, cell-sized lipid vesicles facilitate the observation of membrane dynamics [18,19,20,21,22] and investigation of membrane protein functions [23,24,25,26,27]. Cell-sized lipid vesicles containing biomolecules, enzymes, or cell-free synthesis systems have been used as bioreactors or artificial cell models to understand cellular functions or the “origin of life” [28,29,30,31,32,33]. Artificial or minimal cell models are assembled from biomolecules like phospholipids, protein, and DNA. One approach for the assembly of minimal or artificial cell models from biomolecules is the bottom-up approach [34,35,36,37]. In this approach, membrane proteins are expressed and purified from living cells like *Escherichia coli (E. coli)*, yeast, insect, and mammalian cells, and reconstituted into giant lipid vesicles. The elemental functions of the membrane proteins are then investigated in the giant vesicles. Using this method, the binding affinity of various chemicals to G-protein-coupled receptors (GPCR) on the vesicle membrane has been investigated. Transporters integrated into lipid vesicles facilitate the transport of ions or small molecules across membranes. Using cell-sized vesicles containing cell-free synthesis systems, various types of proteins have been expressed in the cell-sized vesicles and artificial cell models like ion channels, enzymes, receptors, and cytoskeleton proteins created by interactions between the functional proteins expressed by the cell-free synthesis systems.

Recently, microfluidic technology has been used for the development of well-defined cell-sized lipid vesicles [38,39,40,41,42,43,44,45]. The growth of synthetic biology has enabled the expression, purification, and reconstitution of various protein types owing to the development of complex artificial cell models [46,47,48,49]. The giant lipid vesicles generated using microfluidic technology are monodispersive vesicles with high encapsulation efficiency. The distribution of the asymmetric lipid bilayer mimics that of the plasma membrane in eukaryotic cells. Various proteins from different species and with diverse functions have been reconstituted into single giant lipid vesicles as biological elements. For example, the molecules in the outer solution that receive information from sequential biological reactions have been formed into single giant lipid vesicles. In this review, the formations of giant lipid vesicles that emulate living cells, using microfluidic technology, and the cell functions of sequential complex protein reactions into the giant lipid vesicles have been discussed.

## 2. Conventional Methods of Giant Lipid Vesicle Formation

The gentle hydration method is the most straightforward method of giant lipid vesicle formation [50,51]. Although this method was developed in the 1960s, numerous researchers have used giant lipid vesicles formed by the gentle hydration method [18,52,53]. In this method, a low concentration (about 1 mM) of phospholipids dissolved in chloroform are transferred to a glass microtube and dried under a flow of argon gas, leading to the formation of phospholipid films on the glass surface. These films are hydrated using pure water or buffer solutions and incubated for a few hours at 25 °C, resulting in the swelling of the phospholipid films and leading to the formation of giant lipid vesicles. The size distribution of giant lipid vesicles formed by this method is wide (ranging from a few micrometers to over 100 μm).

## 3. Formation of Giant Lipid Vesicles Using Microfluidic Technologies

Although the conventional methods, such as the gentle hydration method, can help in the formation of a large amount of giant lipid vesicles, these vesicles do not achieve the monodispersive, unilamellar encapsulation of high concentration and formation of an asymmetric lipid bilayer. The plasma membrane of eukaryotic cells contains an asymmetric lipid bilayer [54,55,56,57,58,59]. The outer leaflet of the plasma membrane mainly consists of phosphatidylcholine (PC) and sphingomyelin (SM), and the inner leaflet mainly consists of phosphatidylserine (PS) and phosphatidylethanolamine (PE). On the other hand, the outer leaflet of the outer membrane of *E. coli* consists of carbohydrate chain-integrated phospholipids [60,61]. Therefore, asymmetric lipid distribution plays a critical role in recognizing normal cells or apoptosis cells.

Microfluidic technologies for droplet formation using a T-junction device or flow-focusing device achieve the monodispersive and highly concentrated encapsulation. Therefore, to improve the monodispersive, unilamellar, highly concentrated encapsulation and asymmetric lipid distribution of the giant lipid vesicles, microfluidic technologies for giant lipid vesicle formations have been developed (Table 1).

### 3.1. Droplet Transfer Method

**Figure 1:**
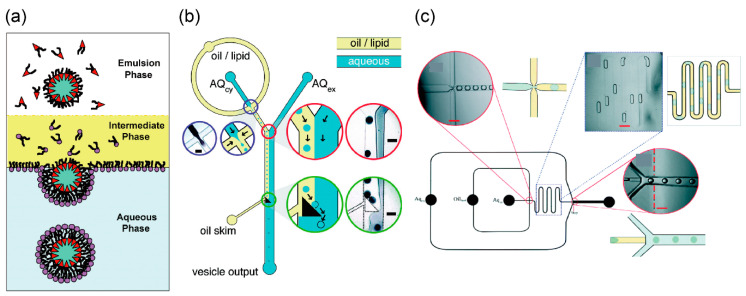
(**a**) Formation of asymmetric lipid vesicles by droplet transfer method. Reproduced with permission from [63]. (**b**,**c**) Microfluidic devices of cell-sized lipid vesicle formations based on the droplet transfer method. Reproduced with permission from [66,70]. The water-in-oil (w/o) emulations were gangrened by a flow-focusing technology. The giant lipid vesicles were formed by the phase transfer (from the oil/lipid phase to the aqueous phase) of the w/o emulations.

The droplet transfer method for the development of asymmetric lipid vesicles was first reported by Pautot et al. (Figure 1a) [62,63]. First, the buffer solution (forming the inner solution of giant vesicles) is added to a solution of phospholipids dissolved in an organic solvent like mineral oil, and a water-in-oil (w/o) emulsion is formed by pipetting or vortexing. The buffer solution (forming the outer solution of giant vesicles) and phospholipid solutions that form the w/o emulsions appear stratified when applied to a microtube. By gravity, the w/o emulsions are transferred to the interface between the phospholipid and buffer solutions, thus forming the outer solution of the giant vesicles. Consequently, the giant vesicles with the phospholipid bilayer are formed from the w/o emulsions. During the generation of asymmetric lipid vesicles by the droplet transfer method, the components of the phospholipid monolayer of the phospholipid–water interface are different from the phospholipids in the w/o emulsion. The giant lipid vesicles formed using the droplet transfer method are more monodispersive, unilamellar, and encapsulated, compared to the giant lipid vesicles formed using the gentle hydration method. These vesicles are formed in 10–20 min owing to the presence of organic solvent (*n*-decane) between the lipid monolayers. Giant lipid vesicle formation methods using microfluidic devices, which are integrated in the droplet transfer method, have been reported (Figure 1b,c) [64,65,66,67,68,69,70,71,72]. The microfluidic technologies of the droplet transfer method have been used for the generation of the w/o emulsions and the phase transfer (from the oil/lipid phase to aqueous phase) of the w/o emulsions.

### 3.2. Asymmetric Lipid Vesicles Formation Using Microfluidic Technology

Funakoshi et al. developed a pulsed-jet flow method for giant lipid vesicle formation [73]. In this method, the giant lipid vesicles are generated by applying a pulsed-jet flow to a planar lipid bilayer, which is formed by the droplet contact method by contact of the lipid monolayers with the w/o droplets in an infinity-shaped chamber. The pulsed-jet flow is generated by a commercial microdispenser placed between a glass capillary nozzle and an air compressor.

The planar lipid bilayer formed by the droplet contact method is used to measure ion channel currents. To measure the ion current of nanopores or ion channels on the planar lipid bilayer, electrodes are assembled at the bottom of infinity-shaped chambers connected to a patch clamp amplifier [74]. Multiple planar lipid bilayer devices have been developed to comprehend the drug screening process of ion channels [75,76]. This system has been used to measure signals from biological nanopores and various types of ion channels expressed on the plasma membrane or other organelle membranes in living cells [77]. Additionally, small-molecule single-strand DNA or miRNA has also been detected using biological nanopores [78,79,80,81,82].

This vesicle formation method using the pulsed-jet flow can be used to encapsulate mammalian cells into giant vesicles. However, the formation of these vesicles takes 10–20 min owing to the presence of an organic solvent (*n*-decane) between the lipid monolayers (this vesicle is a water/oil/water vesicle) [73]. To form an asymmetric lipid vesicle without the organic solvent layer, the pressure and application time of the jet flow need to be raised. Stachowiak et al. generated a stable giant vesicle of approximately 200 μm in diameter using a pulsed-jet flow applied with a piezoelectric actuator. This method can also be used to encapsulate fluorescent beads or small lipid vesicles of 500 nm diameter into giant vesicles [83,84]. Asymmetric lipid vesicles can be generated by fusing small lipid vesicles (about 100 nm in diameter) to a planar lipid monolayer [85]. In addition, membrane proteins can be reconstituted into giant vesicles by fusing membrane protein-reconstituted small vesicles to a planar lipid monolayer. The authors have observed the fusion of small vesicles encapsulated into SNARE-reconstituted giant vesicles. Recently, claudin-4 was reconstituted into a giant vesicle using this method and the membrane adhesion was brought about by mediating claudin-4 interactions [86].

**Figure 2:**
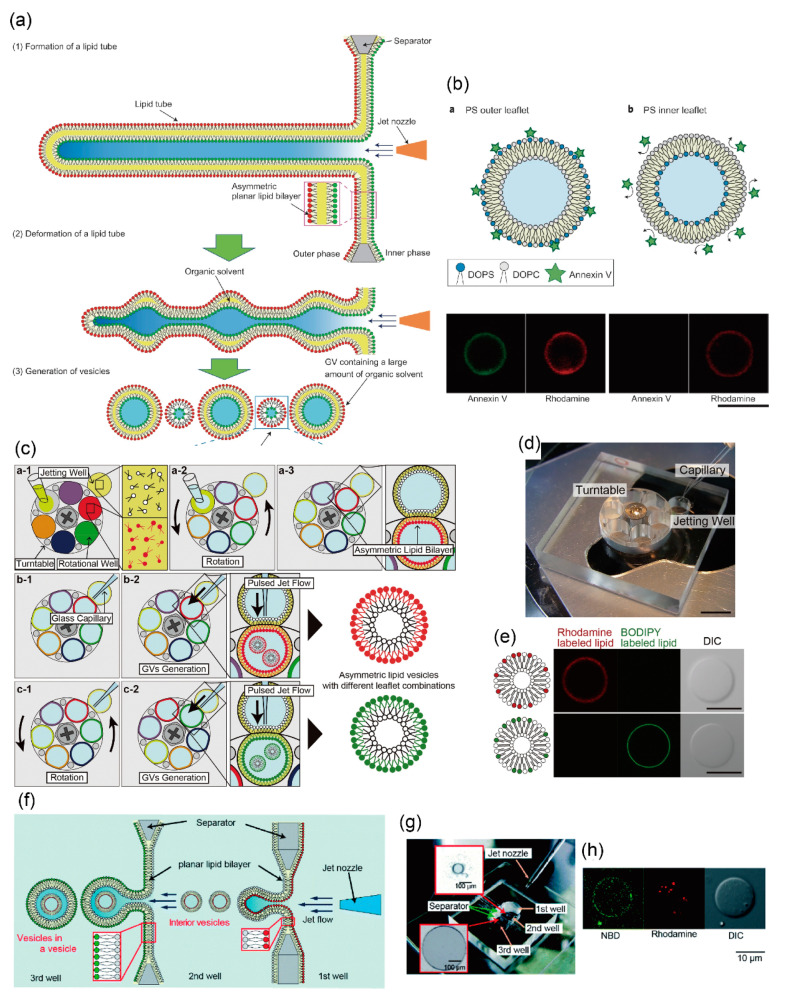
(**a**) Cell-sized asymmetric lipid vesicles from a planar asymmetric lipid bilayer using the pulsed-jet flow; (**b**) asymmetric lipid vesicles with DOPS on the outer leaflet or the inner leaflet. The green fluorescence represents annexinV and the red fluorescence represents the lipid membrane (rhodamine). ((**a**,**b**) are reproduced with permission from [87].). (**c**) Schematic images of a sequential giant lipid vesicle generation method with various lipid combinations using a rotational well device; (**d**) image of the fabricated device for generating sequential cell-sized asymmetric lipid vesicles. The device consists of a turntable with six wells and a base with one jetting well; (**e**) typical confocal images of cell-sized asymmetric lipid vesicles with rhodamine or BODIPY-conjugated lipid on the outer leaflet using the rotational well device. ((**c**–**e**) are reproduced with permission from [88].). (**f**) Schematic illustration of vesicles-in-a-vesicle generation using pulsed-jet flow method. (**g**) Image of a triple-well device for generating vesicles-in-a-vesicle; (**h**) typical confocal images of vesicles-in-a-vesicle using the triple-well device. Small vesicles with rhodamine-conjugated lipids were encapsulated into giant vesicles with NBD-conjugated lipids. ((**f**–**h**) are reproduced with permission from [90].).

In another method for generating asymmetric lipid vesicles using jet flow, Kamiya et al. directly prepared the asymmetric planar lipid bilayer in infinity-shaped wells. On application of the pulsed-jet flow (application time: 2–6 ms, and application pressure: 300 kPa), the asymmetric lipid bilayer, a lipid microtube, was formed, and two vesicles approximately 150 and 5–20 μm in diameter were subsequently generated by the deformation of the lipid microtube. Large-sized vesicles of about 150 μm in diameter were included in the organic solvent (*n*-decane) layer present between the lipid bilayers, while small-sized vesicles of about 5–20 μm in diameter were not contained in the *n*-decane layer (Figure 2a,b) [87]. The small-sized vesicles of approximately 5–20 μm in diameter were found to be stable for more than seven days. Asymmetric lipid vesicles can be used to observe the dynamics of the lipid flip-flop phenomenon and the interactions between lipids and peptides. The amount of connexins reconstituted into giant lipid vesicles is different from that of the asymmetric lipid components. Therefore, asymmetric lipid vesicles facilitate the observation of the functions of membrane proteins present in the asymmetric lipid bilayer, and the formation of well-defined artificial cell models.

Recently, Gotanda et al. developed a device to sequentially generate asymmetric lipid vesicles using various combinations of asymmetric lipid membranes. Various types of asymmetric planar lipid bilayers were formed by establishing contact between the jetting well and the rotational well by rotating a turntable with six rotational wells (Figure 2c,d) [88]. Various compartments of asymmetric lipid vesicles were generated by applying jet flow to various combinations of the asymmetric planar lipid bilayers. The mechanical motion of the planar lipid bilayer and the giant lipid vesicle, including rotation of the turntable and application of the pulsed-jet flow, did not lead to the exchange of lipids between the outer and inner leaflets (Figure 2e). Moreover, to achieve a constant speed of rotation, the turntable was rotated using a stepping motor. The optimum parameters for rotational speed to produce a highly planar lipid bilayer was estimated. Giant lipid vesicles were automatically generated by controlling the speed and timing of rotation of the turntable and application time of the jet flow, under a microcomputer [89]. This automated system of giant lipid vesicle generation involves a high-throughput assay for interaction between the biomolecules (membrane proteins, peptides, and lipids) and various compartments of the asymmetric lipid membranes.

Using this pulsed-jet flow method, Kamiya et al. developed a cell-sized asymmetric lipid vesicle containing small-sized asymmetric lipid vesicles (asymmetric vesicles-in-a-vesicle) [90]. This asymmetric vesicles-in-a-vesicle mimicked the asymmetric lipid distribution of the plasma membrane and other intracellular vesicle membranes of eukaryotic cells (Figure 2f). The intracellular vesicles consist of phosphatidylserine (PS) and phosphatidylethanolamine (PE) on the outer leaflet and phosphatidylcholine (PC) and sphingomyelin on the inner leaflet. The lipid distribution of the intracellular vesicle membranes is different from that of the plasma membrane. The asymmetric vesicles-in-a-vesicle are generated by applying a pulsed-jet flow against two parallel planar lipid bilayers in a triple-well device (Figure 2g,h).

Asymmetric lipid vesicles have been developed using different microfluidic devices. Hu et al. developed asymmetric lipid vesicles from lipid droplets generated in the microfluidic device [65]. Water droplets (w/o emulsions) of various diameters (from less than 10 μm to greater than 120 μm) were generated in a T-shaped microfluidic device. Asymmetric lipid vesicles with different lipids on the outer and inner leaflets were generated by forming the outer leaflet lipid monolayer on the water–organic solvent interface in the microtube. Asymmetric lipid vesicles of various diameters were generated by the phase transfer of water droplets from the organic solvent phase to the water phase by centrifugal force. These asymmetric lipid vesicles contain rhodamine- or biotin-conjugated phospholipids, or phosphatidylserine on the inner and outer leaflets. The asymmetric lipid vesicles formed by this method were stable for 68 h at 4 °C. However, residual organic solvent was observed in the lipid bilayer.

**Figure 3:**
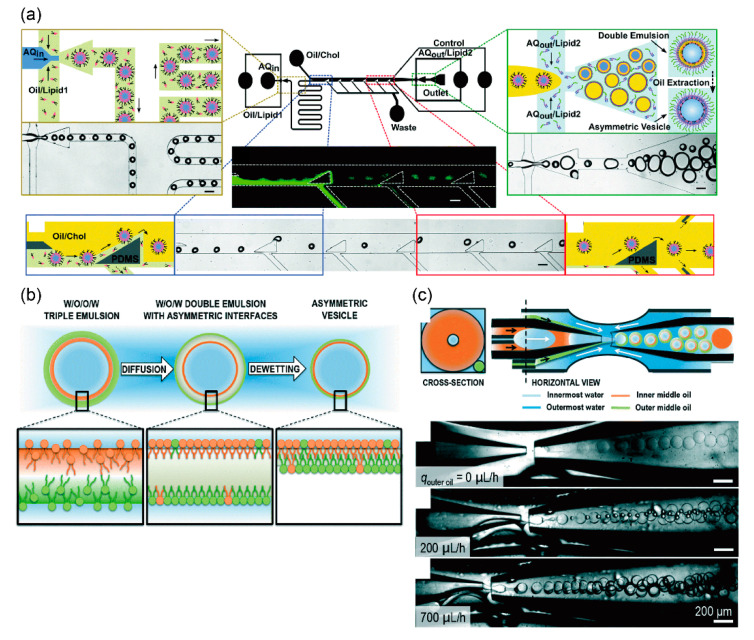
(**a**) Microfluidic device for generating asymmetric water-in-oil-in-water double emulsions with 50–150 µm diameter. Asymmetric lipid vesicles are generated from the asymmetric double emulsions by extracting an intermediate oil layer. Reproduced with permission from [98]. (**b**) Schematic illustration of a formation method of asymmetric lipid vesicles generated from water-in-oil-in-oil-in-water triple emulsions; (**c**) illustration and optical microscope images of microfluidic devices to form the triple emulsions. ((**b**,**c**) are reproduced with permission from [99].).

The giant lipid vesicles from the de-wetting of a water-in-oil-in-water (w/o/w) double emulsion were prepared using a coaxial microcapillary fluidic device [91,92,93,94,95,96]. Recently, Chiarot et al. also developed asymmetric lipid vesicles in a different microfluidic device (Figure 3a) [97]. First, uniform water-in-oil (w/o) emulsions were generated by the flow focusing method. To produce asymmetric lipid bilayers, the oil/phospholipid solution of the inner leaflet was replaced by other oil/phospholipid solutions of the outer leaflet in a microchannel. Next, w/o/w double emulsions were generated by the flow focusing method. Lipid vesicles were formed from the w/o/w emulsion by de-wetting. The diameter of the giant vesicles formed by this method was about 60 μm. The giant vesicles formed were highly monodispersive. The asymmetric lipid vesicles were contained in nitrobenzoxadiazole (NBD)- and Texas red-phospholipids on the outer and inner leaflets, respectively. Asymmetric lipid vesicles containing lipopolysaccharide (LPS) on the outer leaflet and DOPC/DPPC/cholesterol on the inner leaflet were generated using the microfluidic device [98]. These vesicles mimic the outer membranes of *Pseudomonas aeruginosa.* The bending and area expansion moduli of the asymmetric lipid bilayer were investigated.

The Weitz group developed asymmetric lipid vesicles by using water-in-oil-in-oil-in-water (w/o/o/w) triple-emulsion drops to direct the assembly of the two leaflets [99]. These triple-emulsion drops were generated by a device modified by a coaxial microcapillary fluidic device. The triple-emulsion drops have two ultra-thin oil shells, both made of the same oil but differing in their lipid composition. Consequently, w/o/w double-emulsion drops with asymmetric interfacial composition were formed spontaneously. De-wetting of the middle oil phase from the innermost water core of the double emulsion induced the formation of a vesicle bilayer with asymmetric lipid composition (Figure 3b). The asymmetricity ratio of this method was approximately 70%. The asymmetric lipid vesicles formed by this method are stable for over 24 h. High-throughput production (200 vesicles/s) can be achieved by this method and the vesicles formed by this method are highly monodispersed drops (Figure 3c).

## 4. Development of Giant Vesicles with Complex Shapes Using Microfluidic Technology

The formation of giant lipid vesicles depends on self-assembly methods like the gentle hydration method and the electroformation method. These methods generate spherical-unilamellar vesicles. Giant vesicles of various complex shapes like layer-by-layer, multi-compartment, or vesicles-in-a-vesicle have been generated by the top-down approach using microfluidic technology [42,100,101]. The Paegel group developed a layer-by-layer (double bilayer) lipid vesicle using a microfluidic device [102]. First, w/o emulsions were generated in the organic solvent using the capture cups in the microfluidic device. Phospholipids dissolved in the organic solvent and aqueous solution were replaced in the microfluidic device, and asymmetric lipid vesicles were formed in the capture cups. The asymmetric layer-by-layer lipid vesicles were formed by repeating this process. These layer-by-layer vesicles are useful for investigating the complex cell membranes, and other organelle membranes like nuclear envelopes and mitochondrial membranes.

**Figure 4:**
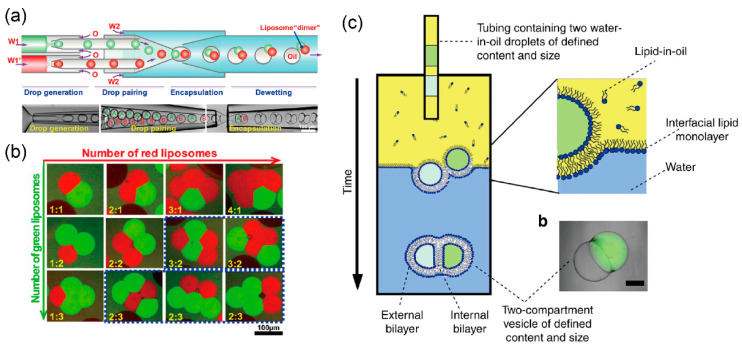
(**a**) Formation of double emulsions with two distinct drops into the microfluidic device; (**b**) control of the structure and the number of compartments of cell-sized lipid vesicles. ((**a**,**b**) are reproduced with permission from [103].). (**c**) Multicompartment giant lipid vesicles formed by expelling the w/o droplets from the capillary; reproduced with permission from [105].

The Huck group developed a multi-compartment lipid vesicle using a coaxial microcapillary fluidic device [103,104]. De-wetting of the w/o/w emulsions led to the assembly of multi-compartment lipid vesicles from these emulsions, which contain few w/o emulsions generated by a coaxial microcapillary fluidic device (Figure 4a). The number of compartments can be controlled by changing the flow rate of the independent droplet generator (Figure 4b). These vesicles are used in vesicle networks that mimic cell–cell communications.

The Ces group generated a multi-compartment lipid vesicle for sequential reactions by expelling multiple w/o droplets from the capillary tube using the phase transfer method (Figure 4c) [105]. Sequential enzyme reactions occurred in each compartment of the lipid vesicle. Therefore, these multi-compartment lipid vesicles serve as micro-reactors for producing biological or chemical products that are difficult to produce in living cells.

Weiss et al. developed a microfluidic technology to generate stable, definite-sized liposomes termed as “droplet-stabilized giant unilamellar vesicles (dsGUVs)” [106]. dsGUVs enable the sequential loading of biomolecules including transmembrane and cytoskeleton proteins by microfluidic pico-injection technology. The dsGUVs merge lipid vesicles and copolymer-stabilized droplets mechanically and chemically to generate stable cell-like compartments. GUVs are formed in copolymer-stabilized droplets containing large unilamellar vesicles (LUVs), thereby inducing membrane fusion in the presence of 10 mM Mg^2+^. To inject biomolecules from a pico-injection channel (injection volume ranges between 2 and 100 pL) to the aqueous phase of the dsGUV, an alternating electric potential (1 kHz and 250 V) was applied to the contact area between the surface of the dsGUV and the pico-injection channel. In this system, integrin or ATP synthase (transmembrane proteins)-containing proteoliposomes or cytoskeletal proteins (soluble proteins) were encapsulated in the dsGUV and fused with the lipid bilayer in the stabilizing polymer droplets. The GUVs, including biomolecules, were released from the surrounding stabilizing polymer droplets into the aqueous phase using the microfluidic device. Following injection of the dsGUV into the microfluidic device, the dsGUVs were separated from each other at the T-junction where a tributary oil flow containing 20% vol. destabilizing surfactant merged with the droplet flow. Passive trapping structures within the microfluidic channel enabled the draining of the continuous oil phase into adjacent oil outlets and decelerated the droplets before they entered the aqueous phase. The functions of integrins, ATP synthases, and actins in the dsGUV, and the release of the GUV were observed using fluorescence microscopy. The formation of synthetic cells using microfluidic technology is a high-throughput method enabling the generation of up to 10^3^ functional compartments each second.

## 5. Artificial Cell Models Using Giant Lipid Vesicles

Giant lipid vesicles comprising the lipid bilayer mimic the cell membrane in terms of structure, size, and biophysical or biochemical properties. Cell-sized lipid vesicles can be easily observed using an optical microscope. Therefore, giant lipid vesicles are used as artificial cell models. Enzymes, substrates, and small biomolecules are encapsulated into the giant lipid vesicles for emulating or observing individual functions of the cytoplasm of living cells. Membrane proteins containing ion channels, transporters, receptors, and enzymes are reconstituted in the giant lipid vesicles to observe the communication between the outer and inner environments of living cells. Until recently, one kind of biomolecule was known to express only one kind of function in the giant lipid vesicle. Improvements in the reconstitution technique of biomolecules or the production method of giant lipid vesicles has facilitated the generation of artificial cell models with multiple functions by the reconstitution of a large number of biomolecules in the giant lipid vesicles. The field of synthetic biology has helped study protein expression using cell-free synthesis systems.

### 5.1. Biological Compartments for Producing Artificial Cell Models

#### 5.1.1. Pore-Forming Protein

Pore-forming proteins are important for the exchange of biomolecules and ions between the inner and outer environments of giant lipid vesicles [107]. They are of various sizes and facilitate the uptake of energy and ions for maintaining and stimulating biological and chemical reactions in the giant lipid vesicles, respectively. Certain types of pore-forming proteins have specific functions like ion transport or controlling the opening and closing of nanopores by the simulation of light, membrane surface tension, and small molecules. In the field of artificial cell membranes, one of the most famous nanopores is α-hemolysin (αHL), which forms a pore of 1.4 nm in diameter [108]. αHL is easily reconstituted in the lipid bilayer and passes through with the ions and single-stranded DNA. Therefore, αHL makes use of biosensors for sensing single-stranded DNA, microRNA, and small molecules [107,109,110]. The expression of proteins of cell-free synthesis systems in giant lipid vesicles stopped within 5 h owing to the exhaustion of amino acids or energy (ATP and GTP). In the case of reconstitution of αHL in the giant lipid vesicle, the amino acids, as well as energy, were supplied to the inner aqueous phase of these vesicles though the αHL nanopores [111]. Therefore, the protein expression of cell-free synthesis systems continued for prolonged periods of time in giant lipid vesicles with αHL nanopores. The molecular transport of ions, fluorescent dyes, and single-strand DNA through αHL nanopores has been used by various researchers in artificial cell models.

Streptolysin O from hemolytic streptococcus is a pore-forming protein. The pore formation in streptolysin O is induced by the cholesterol-containing lipid bilayer. Polymerized streptolysin O on the lipid bilayer forms nanopores with a diameter of up to 30 nm [112]. The nanopore of streptolysin O is larger and more specific compared to that of αHL. In mammalian cells, large-molecular-weight molecules (<2000 kDa) were delivered into the cell via streptolysin O nanopores on the plasma membranes, and the plasma membranes were then resealed by adding calcium ions and rapidly repairing the injured membranes [113]. Using this cell reseal method in streptolysin O nanopores, the timing of biomolecule transport can be controlled.

Ionophores, some of which are antibiotics, are composed of smaller molecules (approximately 1000 kDa) when compared to nanopores [114]. They are reconstituted in lipid bilayers like cell membranes and liposomes, and they increase the permeability of ions across these lipid bilayers. Most ionophores are involved in specific ion transport [115]. For instance, ionomycin from *Streptomyces conglobatus* allows the passage of only calcium ions, valinomycin from *Streptomyces fulvissimus* transports potassium ions selectively, nigericin from *Streptomyces hygroscopicus* transports potassium and hydrogen ions, monensin from *Streptomyces cinnamonensis* exchanges sodium and hydrogen ions, and gramicidin from *Bacillus brevis* transports cations like potassium, sodium, and hydrogen ions selectively. To allow the selective exchange of ions in/out of the giant lipid vesicles, these ionophores are reconstituted in the giant lipid vesicles [116,117].

An important element of optogenetics is bacteriorhodopsin or channelrhodopsin. This activates the transport of ions by functioning as an ON/OFF switch for light. Bacteriorhodopsin, halorhodopsin, or channelrhodopsin allows the passage of hydrogen, chloride, sodium, potassium, and calcium ions [118]. Recently, the ion transport function of channelrhodopsin, which can be induced by various wavelengths of light, was discovered. The method for the reconstitution of bacteriorhodopsin in giant lipid vesicles has been established. Few authors have reported that the activation and inactivation of biological functions in giant lipid vesicles containing rhodopsin was controlled by light [119,120].

#### 5.1.2. Other Membrane Proteins for Maintaining and Stimulating Reactions

ATP synthase is a transmembrane protein complex responsible for the production of ATP from ADP in a low-pH environment. For instance, ATP synthase was reconstituted in the giant lipid vesicles [121]. The ATP synthase-containing giant lipid vesicles were formed by the rehydration of ATP-synthase-small unilamellar vesicle (SUV) films, and activity of the ATP synthase in the giant lipid vesicles was observed.

The photosynthetic reaction center (RC) is a membrane-spanning protein located in the cell membrane and surrounded by other chlorophyll-based proteins. The RC transduces light energy into a transmembrane pH gradient. To understand the physiological orientation of the RCs expressed in the giant lipid vesicles of *R. sphaeroides*, RC-reconstituted giant lipid vesicles were prepared by the droplet transfer method [122]. The RCs in the w/o droplets of the lipid-rich oil phase were transferred to the aqueous phase by centrifugation. In this method, the RCs were reconstituted in the giant lipid vesicle membranes in a uniform orientation (about 90%) with the dimer of photoenzyme facing the outer aqueous solution.

P-glycoprotein (Pgp, ABCB1), a member of the ATP-binding cassette (ABC) transporter family, is responsible for cellular multidrug resistance against a variety of drugs. P-glycoprotein was reconstituted in small lipid vesicles. The P-glycoprotein-containing small lipid vesicles were deposited or stamped onto ITO glass plates and used for electroformation of giant proteoliposomes [123]. Translocation of the Pgp fluorescent substrate (rhodamine123) across the giant lipid vesicle membranes was observed.

### 5.2. Protein Expression in Giant Vesicles Using Cell-Free Protein Synthesis Systems

Cell-free protein synthesis systems are one of the most important technologies for the construction of artificial cell models by the bottom-up approach [124]. Cell-free protein synthesis has certain advantages. It creates a versatile reaction condition in terms of salt concentration, addition of other compounds, incubation temperature, and toxic products for host cells, for directly assessing the reaction environment by measuring the protein activity without purification, and for long-term reactions compared to host cells. Recently, cell-free protein synthesis systems of crude cell extracts have been obtained from *E. coli*, rabbit reticulocyte [125], wheat germ [126], *Spodoptera frugiperda* [127], and mammalian (CHO or Hela) cells [128,129]. Protein synthesis using recombinant elements (PURE) is a minimal-synthesis system that uses a set of purified elements for the translation reaction [130]. Using these cell-free synthesis systems, various types of proteins including molecular weights, soluble proteins, and membrane proteins can be expressed. The cell-free synthesis system and template DNA or RNA are encapsulated into giant lipid vesicles formed by the droplet transfer method or the microfluidic-based vesicle formation method to create artificial cell models for emulating the metabolism in giant lipid vesicles. Soluble proteins such as enzymes are expressed in giant lipid vesicles and act as transducers of biomolecules. Membrane proteins functioning as receptors, ion channels, and transporters expressed by cell-free synthesis systems activate the transport of ions or small molecules, energy synthesis, and recognition of biomolecules on giant lipid vesicle membranes. Generally, to prevent the aggregation of membrane proteins expressed by cell-free synthesis systems, either a detergent (dodecyl-β-D-maltoside (DDM), Triton x-100, Brij, or sodium dodecyl sulfate (SDS)) or an artificial lipid bilayer (nanodiscs, bicelles, or lipid vesicles) is added to the cell-free synthesis systems and DNA under the expression of the membrane proteins. Folded-membrane proteins can be obtained by the selection of species and concentration of the detergent or lipid (charge, critical micelle concentration (CMC) of the detergent).

Ion channels and transporters can be synthesized by cell-free synthesis systems. Kv1.1 and Kv1.3 channels, a family of potassium voltage-gated channels, consist of a tetramer of six-spanning transmembrane proteins. The Kv1.1 and Kv1.3 channels were inserted into 1,2-diphytanoyl-sn-glycero-3-phosphatidylcholine (DPhPC)-based liposome, and the functions of these channels were observed by electrophysiological measurements [131]. A double-spanning mechanosensitive channel of large conductance (MscL) channel, a mechanosensitive channel in the inner membrane of bacteria, can be synthesized using the cell-free synthesis system of *E coli*. The ion channel activity of MscL, measured by the patch-clamp method, was obtained by applying pressure [132]. Connexin 43, which contains four transmembrane domains [133], and a human voltage-dependent anionic channel (hVDAC1) consisting of 19 β strands in the mitochondrial outer membranes [134] were synthesized into the lipid vesicles. Nicotinic acetylcholine receptor (nAChR), a member of the ligand-gated ion channel superfamily, was synthesized using the cell-free synthesis systems and is observed using the patch-clamp method [135]. In summary, various structures and functions of ion channels expressed by cell-free synthesis systems are reconstituted in the artificial lipid bilayer. The ion transport activity of these channels is determined using the patch-clamp method because the analysis of the single ion channel using the patch-clamp method can be conducted by a low concentration of the ion channels.

**Figure 5:**
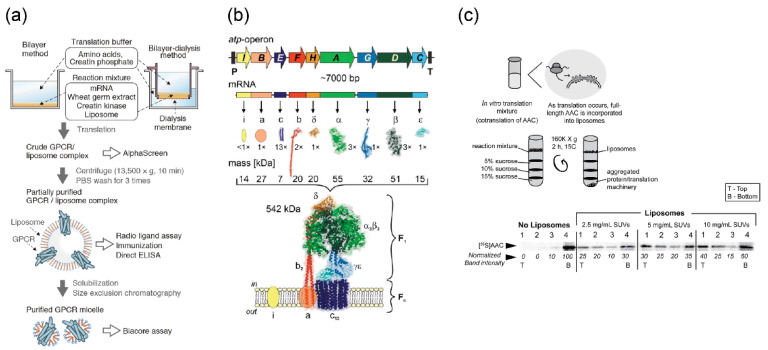
(**a**) Reconstitution of G-protein-coupled receptors (GPCR) expressed by wheat cell-free synthesis system into the lipid vesicles. Reproduced with permission from [137]; (**b**) expression and assembly of a bacterial F_1_F_0_-ATP synthase expressed by *E. Coli* cell-free synthesis into the lipid vesicles. Reproduced with permission from [138]; (**c**) reconstitution of ADP/ATP carrier (AAC) synthesized by wheat cell-free synthesis system into the lipid vesicles. Reproduced with permission from [140].

Membrane proteins other than ion channels and nanopores can also be expressed by cell-free synthesis systems. The G-protein-coupled receptor, which consists of seven spanning transmembrane domains (Figure 5a) [136,137], ATP synthase with complex subunits (Figure 5b) [138,139], and ADP/ATP carrier (AAC), which exchanges ADP and ATP across the mitochondrial inner membranes (Figure 5c) [140], were reconstituted in the lipid vesicles, and their functions or activities were observed.

### 5.3. Giant Lipid Vesicles Containing Multiple Components for Sequential Reactions

To facilitate communication between living and artificial cells or synthetic minimal cells, and the response of the outer environment of the synthetic minimal cells, genetic circuits, and cell-free synthesis systems encapsulated in giant lipid vesicles, and for stimulating the functions of membrane proteins like sensing the outer environment or transporting ions, energy, and small molecules, they are reconstituted in giant lipid vesicle membranes. These synthetic minimal cells cause the assembly of various types of biomolecules using the bottom-up approach. In this section, I have elaborated on synthetic minimal cells containing multiple components.

**Figure 6:**
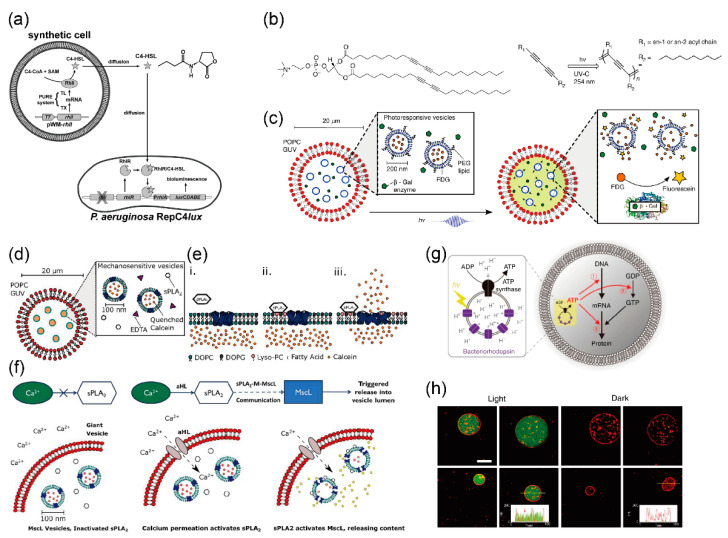
(**a**) Schematic illustration of semi-synthetic minimal cells sending quorum sending signal molecules to *P. aeryginosa*; reproduced with permission from [141]. (**b**) Chemical structure of 1,2-bis(10,12-tricosadiynoyl)-sn-glycero-3-phosphocholine (DC89PC) as photo-responsive lipid; (**c**) schematic illustration of artificial cell models containing nano-sized lipid vesicles by UV-response. The nano-sized DC89PC vesicles containing substrate fluorescein di-beta-D-galactopyranoside (FDG) and the enzyme β-galactosidase are coencapsulated into the giant lipid vesicles. ((**b**,**c**) are reproduced with permission from [144].). (**d**) Schematic illustration of artificial cells containing phospholipase A2 enzyme, EDTA, and mechanosensitive channel of large conductance (MscL) reconstituted into the nano-sized lipid vesicles including quenched calcein. (**e**) Reaction of phospholipase A2 (PLA2) on the nano-sized lipid membranes. (**f**) Synthetic mechanosensitive signaling pathway. Due to α-hemolysin (αHL) into the giant lipid vesicle membranes, calcium ions are transferred to the inner phase of the giant lipid vesicles. The PLA2 function activates the PLA2-membrane-MscL pathway, resulting in content release within the inner phase of the giant lipid vesicles. ((**d**–**f**) are reproduced with permission from [145].). (**g**) Schematic illustration of artificial photosynthetic cells encapsulating artificial organelle containing bacteriorhodopsin and ATP synthase. (**h**) Green fluorescent protein synthesis inside the giant lipid vesicles driven by UV-light. ((**g**,**h**) are reproduced with permission from [146].).

Rampioni et al. developed a synthetic cell to produce and release a chemical signal recognized as “self-signal” by the pathogenic bacterium *Pseudomonas aeruginosa* [141]. Plasmid DNA containing RhlI, the cell-free synthesis system, S-adenosyl-methionine (SAM), and butyryl-coenzyme A (C4-CoA) was encapsulated in the giant lipid vesicles (Figure 6a). RhlI enzymes were synthesized using the cell-free synthesis system in the giant lipid vesicles. The reaction between SAM and C4-CoA, mediated by the enzyme RhlI, produced C4-HSL. The signaling molecules of C4-HSL passed through the lipid bilayer of the synthetic minimal cells and diffused into the medium. The C4-HSL were taken up by the *Pseudomonas aeruginosa* cells. The promoter PrhlA was activated by the RhlR/C4-HSL complex, the luxCDABE operon was expressed, and bioluminescence was emitted by RepC4lux. Thus, the synthetic cells can communicate with living cells and enable the sensing of the environment, signal transduction, and drug production.

Adamala et al. created an encapsulated giant lipid vesicle to enable a modular, controlled compartmentalization of multiple-part genetic circuits and cascades [142]. The synthetic cells proposed by the author enable genetic cascades to proceed in well-isolated environments while permitting the desired degree of control and communication. Synthetic cells containing mammalian transcriptional and translational machinery communicate with other synthetic cells via biological nanopores or by membrane fusion. The synthetic cells encapsulated genetic circuits and the cell extract solution for transcriptional and translational reactions in the giant lipid vesicles, and reconstituted the αHL nanopores on the giant lipid vesicle membranes for the transportation of impermeable activators like isopropyl-β-thiogalactoside (IPTG) and doxycycline (DOX) from the outside of the giant lipid vesicles. Molecules like theophylline and arabinose are permeable to the lipid bilayer. For instance, synthetic cells with bacterial transcription and translation system extract were constructed as communication systems. The sensor liposomes contained IPTG in the non-membrane-permeable activator, which induced the *lac* promoter and the arabinose-inducible gene for αHL. The giant lipid vesicles sensed arabinose and released IPTG by expressing the αHL nanopores. Reporter giant lipid vesicles with αHL nanopores contained a *lac* promoter-controlled firefly luciferase (fLuc). Therefore, the expression of fLuc was controlled either directly (fLuc, under the *lac* promoter) or indirectly (T7RNAP, under the *lac* promoter, and fLuc, under the T7 promoter). Mixing of the inner phase of the giant lipid vesicles by the fusion of the giant lipid vesicle population A and the giant lipid vesicle population B using SNARE proteins induced the reactions of genetic circuits and cascades. The synthetic cells reported by the author enable the study of the more complex characteristics and the mechanisms of Darwinian evolution.

To dynamically control chemical reactions like ATP synthesis or couple them with other intravascular reactions like metabolic reactions, Lee et al. developed synthetic cells using switchable photosynthetic nano-sized lipid vesicles (~100 nm) as energy modules and ATP synthase for producing ATP from ADP and protons, and reconstituting ionophores in giant lipid vesicles [143]. The switchable photosynthetic nano-sized lipid vesicles have two photoconverters: Plant-derived photosystems II (PSII) and bacteria-derived proteorhodopsin (PR), to induce ATP synthase to catalyze the conversion of ADP to ATP. The absorbance bands for the functional activity of PSII and PR are 440 and 670 nm peaks, and a 520 nm peak, respectively. To apply these different absorbance bands of PSII and PR, the ATP production activity of ATP synthase was controlled. Functional activation of PSII was achieved by irradiation using red light. The protons in the nano-sized lipid vesicles were generated by PSII activity and low pH in the vesicles, and ATP synthase activity was induced, leading to ATP production. Consequently, the conversion of ADP to ATP outside the nano-sized lipid vesicles was caused by ATP synthase. When irradiated by green light, PR, which transports protons from the inside of the nano-sized lipid vesicles to the outside, was activated. Consequently, the pH of the solution in the inner phase of the nano-sized lipid vesicles increased and the activity of ATP synthase was inhibited. Using this synthetic cell model, ATP synthesis can be facilitated or impeded by the stimulation of PSII and PR with red or green light, respectively. Finally, sufficient ATP concentration in the giant lipid vesicles achieved by ATP production in the nano-sized lipid vesicles resulted in actin polymerization in the presence of magnesium ions, which passed through magnesium ionophores into the giant lipid vesicles. To control actin interaction, giant lipid vesicles with phase separation membranes were generated: A liquid-disordered phase composed of unsaturated phospholipids that produce a strong attractive interaction, and a liquid-ordered phase composed of sphingomyelins and cholesterols that produce a weak attractive interaction. The difference between actin interactions in the local membrane induced changes in the global cell curvature, deforming the spherical cells into mushroom-shaped cells. Introducing networks of proteins and organelles into artificial cell-like environments may contribute to the achievement of building a cell de novo.

The Ces group created an artificial cell model of cell-sized lipid vesicles containing nano-sized lipids (vesicles-in-a-vesicle), where the inner compartment acted as a phototransducer, responding to UV irradiation through 1,2-bis(10,12-tricosadiynoyl)-sn-glycero-3-phosphocholine (DC_89_PC) polymerization-induced pores (Figure 6b). Bata-galactosidase (bata-gal) and nano-sized lipid vesicles containing fluorescein di-beta-D-galactopyranoside (FDG) were encapsulated in the giant lipid vesicles formed using the droplet transfer method [144]. Reconstitution of DC_89_PC in the lipid bilayer induced photo-polymerization of diacetylene molecules in response to UV irradiation. DC_89_PC-polymerization resulted in the creation of pores, and FDG, which was encapsulated in the nano-sized lipid vesicles, was released to the aqueous phase of the giant lipid vesicles. In the presence of beta-gal and FDG in the giant lipid vesicles, the non-fluorescent fluorogenic substrate FDG was hydrolyzed by the enzyme beta-gal, and fluorescein was illuminated in the giant lipid vesicles (Figure 6c). In this artificial cell model, nanopore formation was controlled by responding to UV irradiation, which enabled the controlled mixing of small molecules between the two comportments.

The Ces group also developed an artificial cell model including a de novo synthetic signaling pathway encapsulated in the vesicles-in-a-vesicle. Secretory phospholipase A2 (sPLA2) and nano-sized lipid vesicles containing high concentrations of calcein, a mechanosensitive channel of large conductance (MscL), were reconstituted in the giant lipid vesicles (Figure 6d,e) [145]. First, calcium ions were transported into the aqueous phase of the giant lipid vesicles through αHL (Figure 6f). In the aqueous phase of the giant lipid vesicles, sPLA2, which is a calcium-dependent enzyme, catalyzed the conversion of phosphatidylcholine to lyso-phosphatidylcholine (LPC) and a concomitant fatty acid at the sn-2 position. Owing to the enzyme activity of sPLA2, the phosphatidylcholine on the outer leaflet of the nano-sized lipid vesicles was converted to LPC. The generation of LPC in the outer leaflet of the nano-sized lipid vesicles resulted in an asymmetric change in the lateral pressure profile of the lipid membranes. MscL responds to changes in the membrane pressure by opening a large nanopore ~2.5–3 nm in diameter, resulting in the release of molecules up to 10 kDa (Figure 6e). Subsequently, a high concentration of calcein was released from the nano-sized lipid vesicles to the aqueous phase of the giant lipid vesicles, leading to the emission of green fluorescence into the aqueous phase of the giant lipid vesicles. Various proteins like αHL, MscL, and sPLA2 are contained in this artificial cell model. Therefore, the advantage of constructing artificial cell models based on giant lipid vesicles using the bottom-up approach and synthetic biology is that the elements of existing biological machinery can be combined with non-native molecular systems.

The Kuruma group developed a light-induced artificial cell model using functional nano-sized lipid vesicles encapsulated in the giant lipid vesicles [146]. To produce ATP by inducing light, bacteriorhodopsin and ATP were reconstituted in the nano-sized lipid vesicles (Figure 6g). The ATP generated in the aqueous phase of the giant lipid vesicles was used as the substrate for mRNA, energy for phosphorylation of guanosine diphosphate (GDP), and aminoacylation of transfer RNA. The author thus demonstrated green fluorescent protein (GFP) fluorescence in giant lipid vesicles by irradiating light (Figure 6h).

## 6. Conclusions and Future Directions

In this review, methods for the development of asymmetric lipid vesicles that mimic eukaryotic cells in terms of their phospholipid distribution, and complex-shaped lipid vesicles based on microfluidic technologies, were elaborated. Moreover, the complex functions of giant lipid vesicles including the stimulation of various types of biomolecules by light or the outside environment of the giant lipid vesicles were also described.

Microfluidic technology allows for the formation of monodispersive giant lipid vesicles with highly encapsulated biomolecules and asymmetric lipid distribution. It also facilitates the formation of well-defined giant lipid vesicles. Although the formation of giant lipid vesicles with these properties such as monodisperse, high encapsulation, and asymmetric lipid distribution is difficult using traditional methods like the gentle hydration method and electroformation method, the production of a large amount of giant lipid vesicles is by the traditional formation method compared to microfluidic technology. Therefore, to increase the amount of giant lipid vesicles produced by microfluidic technology, the traditional methods will be integrated into an automation gimmick. A nuclear membrane and a mitochondrial membrane have unique membrane structures and complex membrane shapes. Currently, the emulation of these organelle membranes using microfluidic technologies is difficult. The lipid membranes that emulate the organelle shapes may be generated around the artificial cytoskeletons of these organelles, which are developed using a micro-scale 3D printer. The emulation of the curvature and shape of these organelle membranes will contribute to the understanding of membrane protein functions on these curvature and shape membranes.

To advance the fields of structural biology, biophysics, and biochemistry, various types of proteins including ion channels, nanopores, receptors, and enzymes have been purified from host cells, and the functions of these proteins have been investigated. Giant lipid vesicles reconstituted with proteins and biomolecules enable the imitation of these cellular functions. Recently, artificial cell models with complex sequential reactions have been developed. These artificial cell models were constructed by combining proteins from various species like *E. coli*, Staphylococcus, and humans. The complex sequential reactions in the cells were not constricted by the proteins from the different species. The use of proteins from different species for creating artificial cell models takes the most advantage of giant lipid vesicles.

By combining microfluidic technology with synthetic biology, more well-defined complex artificial cell models can be produced, which will contribute to the production of minimal cells with one function of living cells, like signal transduction, rare biomolecule production, and the presence of metabolic or glycolytic pathways. Communication between artificial and living cells for the stimulation of cell functions or transfer of biomolecules has been established in the artificial cell models or living cells. Therefore, these communications will contribute to the diagnosis of the diseases into the body. Moreover, the production of artificial cell models by the bottom-up approach can produce primitive cells, which will help understand cell evolution.

## Figures and Tables

**Table 1:** Summary of the giant lipid vesicle formations.

Properties	Droplet Transfer Method	Pulsed-Jet Flow Method	Double/Triple-Emulsion Method	Gentle Hydration Method
Unilamellarity	High	High	High	Low
Monodisperse	High	High	Middle	Low
Highly encapsulated	Yes	Yes	Yes	No
Asymmetric membrane	Yes	Yes	Yes	No
Production amount	Middle	Large	Low	Large
Organic solvent	Large	Small	Less	No
Long-term stability	Middle	Yes	Yes	Yes
